# Association of visceral fat area with pre-frailty in Japanese community-dwelling older adults: a cross-sectional study

**DOI:** 10.1186/s12877-022-03377-w

**Published:** 2022-08-19

**Authors:** Ya Su, Michiko Yuki, Natsuka Ogawa

**Affiliations:** 1 Faculty of Health Sciences, Hokkaido University, Kita 12, Nishi 5, Kita-ku, Sapporo, Hokkaido, 060-0812 Japan; 2 School of Nursing, Shanghai Jiao Tong University, 227 South Chongqing Road, Shanghai, 200025 China; 3 Graduate School of Health Sciences, Hokkaido University, Kita 12, Nishi 5, Kita-ku, Sapporo, Hokkaido, 060-0812 Japan

**Keywords:** Visceral fat area, Pre-frailty, Older adults, COVID-19

## Abstract

**Background:**

Screening and intervention in pre-frailty can help prevent or delay frailty among older adults. Being overweight has shown associated with pre-frailty, and overweight is highly prevalent among community-dwelling older adults during COVID-19. However, the impact of visceral fat accumulation remains unclear. This study aimed to explore the association between visceral fat area and pre-frailty in community-dwelling older adults.

**Methods:**

The participants of this study included community-dwelling older adults from three elderly welfare centers. The frailty phenotype was assessed using the frailty screening index. The body composition was measured using bioelectrical impedance analysis.

**Results:**

A total of 214 community-dwelling older adults completed the questionnaire and measurements. After excluding 16 frail participants, 149 (75.3%) were pre-frailty. The mean age of participants was 75.4 ± 5.4 years, and 69.7% (138) of participants were women. There were 54 (27.3%) participants with high visceral fat area. The multivariable model showed that participants with high visceral fat area were at increased risk for pre-frailty (adjusted OR, 3.15; 95% CI, 1.26 − 7.87; *P* = 0.014), even after adjusted for age, sex, health status, and impact of COVID-19 pandemic.

**Conclusions:**

This study suggests that the association between visceral fat accumulation and pre-frailty may help to identify a new target for prevention. Further longitudinal studies are needed to determine their mechanisms in older adults.

## Background

With an aging population, the growing interest in frailty becomes a public health concern. Pre-frailty as a transitional status between robust and frailty had a 50% higher risk of mortality compared with robust older adults [1, 2]. Among Japanese community-dwelling older adults, the prevalence of pre-frailty is 48.1% [3], and the incidence of frailty was significantly higher in pre-frailty older adults [4]. However, in the past two years, the coronavirus disease (COVID-19) pandemic has impacted public health worldwide [5]. Due to physical activity reductions and lifestyle changes [6], measures such as social distancing or staying at home have reported potential to affect muscle mass and negatively impact body fat [7, 8], as well as have the potential to accelerate frailty.

Previous studies have reported that the physical activity time from January to April 2020 was decreased by 36.4% in robust older adults, 33.3% in pre-frailty older adults, and 30.9% in frail older adults, respectively [9]. There were 51.7% of older adults were pre-frailty in January 2020, and 16% of older adults had a new incidence of frailty during the one-year follow-up COVID-19 pandemic period [10]. This may lead to more older adults declining in physical function after the COVID-19 epidemic. Fortunately, pre-frailty is potentially reversible, early screening and intervention in pre-frailty can help prevent, delay or reverse frailty among older adults. Therefore, for successful aging to become a reality in health promotion, a better understanding is required of how pre-frailty might be prevented.

Obesity underlying risk factor for physical inactivity, poor functional performance, several diseases, and mortality among older adults [11, 12]. The previous study has reported obesity defined by visceral fat area (VFA) had the highest performance in discriminating between oldest-old adults with and without mobility disability compared to other common measures of obesity [13]. Visceral fat accumulation, which is a key feature of abdominal obesity, several studies have shown that excess VFA is a well-known risk factor for health [14]. The previous study has shown that overweight/obesity increases the risk for pre-frailty and frailty [15–18]. These studies were defined overweight/obesity by body mass index (BMI), percentage of body fat (PBF), or waist circumference [15, 17]. However, BMI cannot distinguish between lean muscle mass and fat mass, waist circumference cannot distinguish between subcutaneous fat and visceral fat in the abdominal area [13, 19]. Although obesity is known to be associated with physical activity and physical function [20, 21], and pre-frailty is highly prevalent among older adults, it remains unclear about the visceral fat accumulation and pre-frailty. As a better understanding of the relationship between VFA and pre-frailty will be of considerable importance in evolving preventive measures for community-dwelling older adults, this study aimed to explore the relationship between visceral fat and pre-frailty in community-dwelling older adults.

## Methods

### Study design and population

Hokkaido was a region that has been heavily impacted by the COVID-19 pandemic in Japan and was the first region in Japan to declare a state of emergency in late February 2020. There are 10 elderly welfare centers in Sapporo, which are used to promote health, culture, and recreation and are free for those over 60 years old. According to the 2020 national survey that the population of people aged 65 or above in Sapporo, Hokkaido is 549,151 (27.8%). Convenience sampling was conducted to select participants. This study is a descriptive, cross-sectional study that the survey population was recruited in November 2020 in Sapporo, Hokkaido, Japan, from regular attendees of three elderly welfare centers (social facilities for older adults). Participants who met the following criteria were included; (1) aged 65 years or older; (2) able to walk without help; (3) willing to complete the survey; and (4) provided consent to participate. Trained interviewers administered a standardized questionnaire to collect information for the study.

### Ethical approval for studies and informed consent

All the procedures performed in this study were in accordance with the Declaration of Helsinki and were approved by the Ethics Committee of the Faculty of Health Sciences, Hokkaido University (Reference No 20–27). Each participant signed an informed consent document after receiving a detailed verbal explanation of the study objectives.

### Demographic characteristics and self-reported impact of the COVID-19 pandemic

For this study the following information was obtained from the questionnaire: Demographic characteristics included age and sex. Participants were asked whether they were affected during the COVID-19 period: Cannot sleep well (yes/no); Stress and anxiety (yes/no); Going out less than last year (yes/no).

### Frailty

We assessed the frailty phenotype using the frailty screening index, which is a Japanese version of the screening tool based on the Cardiovascular Health Study criteria, the Kihon checklist, and other Japanese questionnaires [22]. The frailty screening index includes five indicators: weight loss, physical function, physical activity, memory loss, and exhaustion. Participants who met three or more of the following five indicators were regarded as frail, and Pre-frailty was defined as meeting one or two of these indicators according to the Cardiovascular Health Study criteria. This frailty screening index is a useful self-report questionnaire for frailty in community-dwelling older adults [22, 23].

### Depression

The 5-item Geriatric Depression Scale (GDS-5) was used to assess depression states among the community-dwelling older adults. This is a short version that comprises 5 questions, a score ≥ 2 suggests the presence of a depressive state [24]. The GDS-5 is a reliable and valid screening tool similar to the 15-item GDS for the screening of depression in older adults [25].

### Anthropometry

BIA has become a simple and useful diagnostic tool for assessing body composition. It utilizes differences in resistance between the fat and lean components of the body to provide an estimate for fat-free mass, total body fat, and VFA. After measuring the height and weight, we measured the body composition using bioelectrical impedance analysis (BIA) (InbodyS10, Biospace, Korea). Participants without a pacemaker were measured in a sitting position, with their arms lowered naturally, and their thighs did not touch each other but stretched to the width of their shoulders. The body composition measurement included BMI, VFA, and PBF. The overweight was defined as BMI (BMI ≥ 25), VFA (VFA ≥ 100) [26], and PBF cutoffs for defining obesity were ≥ 25% for men and ≥ 30% for women [27].

### Statistical analyses

In logistic regression, Pedhazur recommends a ratio of 30 cases for each independent variable included in the model [28]. There were six main variables were measured in this study. Thus, this study purposely selects the 30 cases for each variable, and the total of 180 respondents required in this study fulfills the suggested sample size of logistic regression analysis. All data analyses were conducted using IBM SPSS Statistics Version 26 (IBM, Armonk, New York, USA). Regarding descriptive statistics, continuous variables were presented using mean and standard deviations or median and range, whereas categorical variables using frequencies and percentages. Additionally, differences in characteristics between pre-frailty and robust, VFA ≥ 100 and VFA < 100 were tested with the chi-squared test and Fisher’s exact test for the categorical variables, whereas Student’s t-tests and Mann–Whitney U test were used for the continuous variables. Univariate and multivariate logistic regression analyses were used to test the association between VFA and pre-frailty, with pre-frailty as the dependent variable. Multivariate logistic regression analysis using the following models: Model 1 was adjusted for age and sex, model 2 was adjusted for age, sex, and going out less than last year due to COVID-19, and model 3 was adjusted for age, sex, going out less than last year due to COVID-19, the number of prescription medications taken per day, and depression. Results are presented as odds ratios with 95% confidence intervals. *P*-values of < 0.05 were considered statistically significant. The goodness of fit for multivariate logistic regression models was assessed using the Hosmer‐Lemeshow test (H‐L test). Furthermore, we also used Wilcoxon signed-rank test to analyze the body composition changes before and during COVID-19.

## Results

We recruited 249 participants from three centers in Sapporo. A total of 214 community-dwelling older adults completed the questionnaire and measurements with a response rate of 86%. After excluding 16 frail participants, a total of 198 participants were included are shown in Table 1, of whom 149 (75.3%) were prefrail. The mean age of participants was 75.4 ± 5.4 years, and 69.7% (138) of participants were women. In terms of overweight characteristics, there were no statistically significant differences between pre-frailty and robust groups in PBF, overweight (PBF), and overweight (BMI). However, overweight (VFA) was statistically significant with pre-frailty, moreover, pre-frailty participants had significantly higher BMI and VFA. In terms of health characteristics, depression and the number of prescription medications taken were significant with pre-frailty. Furthermore, in the self-report impact of the COVID-19 pandemic, there were 78% of the older adults have reduced going out due to the COVID-19 pandemic, decrease in going out was significant with pre-frailty.

**Table1:** Association of overweight and impact of the COVID-19 pandemic with pre-frailty

Variables	Total *n* = 198	Robust *n* = 49	Pre-frail *n* = 149	*P* Value	Effect size (*d*)	95% C.I
**Demographic**						
Age	75.4 ± 5.4	74.7 ± 6.0	75.7 ± 5.1	0.316	0.187	-0.136–0.511
Sex (women)	138 (69.7)	36 (73.5)	102 (68.5)	0.508		
**Body composition**						
SMM (kg)	21.03 ± 6.34	20.30 ± 3.73	21.28 ± 4.09	0.178	0.245	-0.079–0.568
SMI (kg/m^2^)	6.34 ± 0.87	6.15 ± 0.83	6.40 ± 0.88	0.087	0.288	-0.036–0.612
BMR (kcal)	1214.94 ± 143.45	1186.92 ± 132.11	1224.15 ± 146.23	0.159	0.260	-0.063–0.584
PBF (%)	30.2 ± 7.1	28.5 ± 7.5	30.8 ± 6.9	0.057	0.326	0.002–0.651
BMI (kg/m^2^)	23.5 ± 3.3	22.4 ± 3.1	23.8 ± 3.2	0.009*	0.441	0.115–0.767
VFA (cm^2^)	81.5 ± 32.8	71.6 ± 28.9	84.8 ± 33.5	0.015*	0.407	0.082–0.732
**Overweight**						
^a^ Overweight (%BF)	127 (64.1)	30 (61.2)	97 (65.1)	0.624	0.070	-0.209–0.349
^b^ Overweight (BMI)	62 (33.6)	12 (24.5)	50 (33.6)	0.235	0.164	-0.110–0.449
^c^ Overweight (VFA)	54 (27.3)	7 (14.3)	47 (31.5)	0.019*	0.338	0.056–0.621
**Health characteristics**						
The number of prescription medications taken per day	2 (0–13)	1 (0–6)	2 (0–13)	0.011*	0.368	0.084–0.651
Depression (GDS5 ≥ 2)	68 (34.3)	11 (22.4)	57 (38.3)	0.043*	0.291	0.009–0.572
**Impact of the COVID-19 pandemic**						
Cannot sleep well	17 (8.6)	4 (8.2)	13 (8.7)	0.903	0.017	-0.261–0.296
Stress and anxiety	50 (25.3)	8 (16.3)	42 (28.2)	0.097	0.238	-0.043–0.518
Going out less than last year	154 (77.8)	31 (63.3)	122 (82.6)	0.005*	0.407	0.123–0.691

Results are presented as mean ± SD, median (range), or n (%)

*Abbreviations:* *SMM* Skeletal Muscle Mass, *SMI* Skeletal Muscle mass Index, *BMR* Basal Metabolic Rate, *PBF* Percentage of body fat, *BMI* Body Mass Index, *VFA* Visceral fat area, *CI* Confidence interval

^a^ Overweight: Men: PBF ≥ 25%, Women: PBF ≥ 30%

^b^ Overweight, BMI ≥ 25 kg/m^2^

^c^ Overweight: Visceral fat area ≥ 100 cm^2^

^*^ *P*-value < 0. 05

**Fig. 1:**
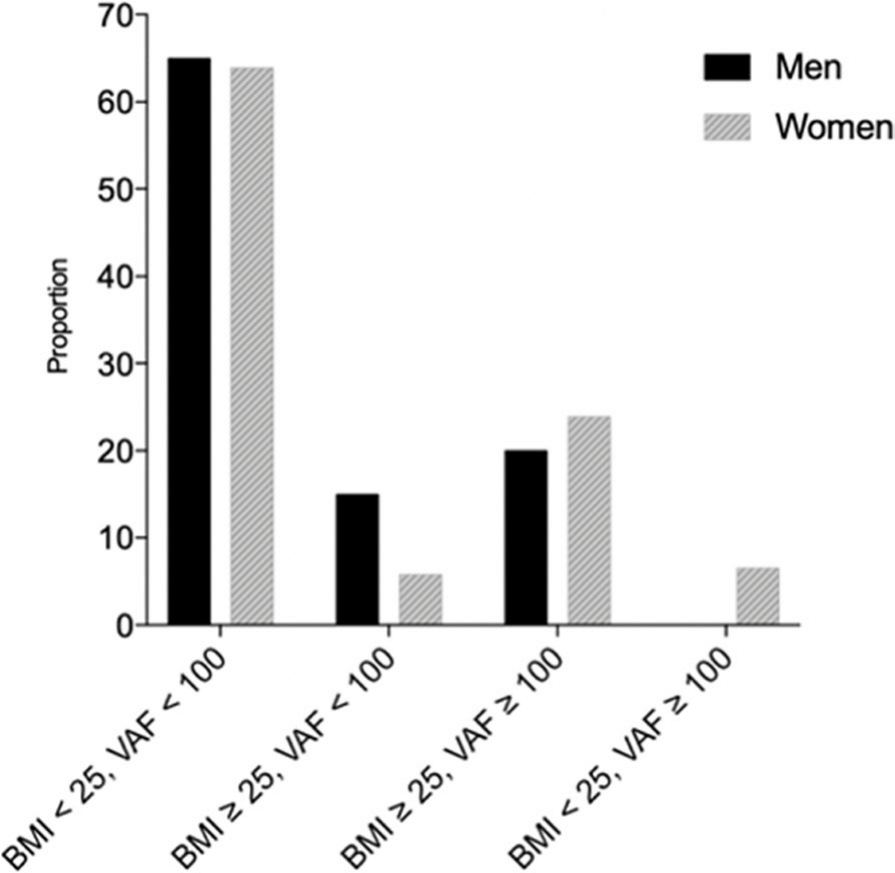
The distribution of BMI and VAF by sex. The following four groups were divided according to BMI and VFA: Non-BMI overweight and non-high VAF; BMI overweight and non-high VAF; BMI overweight and high VAF; non-BMI overweight but have high VAF

The association of VFA with frailty indicators is shown in Table 2. There were 54 (27.3%) participants with the high VFA. The high VFA was significantly associated with low physical function on the frailty screening index. Moreover, it is worth noting that 6.5% of women have no BMI overweight but have high VFA as shown in Fig. 1.

**Table 2:** Association of visceral fat area with frailty indicators

Variables	Total *n* = 198	Visceral fat area		*P* Value
		< 100 cm^2^ *n* = 144	≥ 100 cm^2^ *n* = 54	
**Demographic**				
Age	75.4 ± 5.4	75.7 ± 5.6	74.8 ± 4.6	0.318
Sex (women)	138 (69.7)	96 (66.7)	42 (77.8)	0.130
**Frailty indicators, n (%)**				
Weight loss	30 (15.2)	24 (16.7)	6 (11.1)	0.332
Low physical function	114 (57.6)	75 (52.1)	39 (72.2)	0.011*
Low physical activity	169 (85.4)	126 (87.5)	43 (79.6)	0.163
Memory loss	8 (4.0)	6 (4.2)	2 (3.7)	1.000
Exhaustion	21 (10.6)	12 (8.3)	9 (16.7)	0.090
**Prevalence of pre-frailty, n (%)**	149 (75.3)	102 (70.8)	47 (87.0)	0.019*

Results are presented as mean ± SD, or n (%)

^*^ *P*-value < 0. 05

To explore the association of VFA with pre-frailty we further performed logistic regression analysis for several models, while adjusting for other variables are shown in Table 3. The multivariable-adjusted model showed that participants with VFA ≥ 100 were at increased risk for pre-frailty (adjusted OR, 3.15; 95% CI, 1.26 − 7.87; *P* = 0.014), even after adjusting for health status and impact of COVID-19 pandemic.

**Table 3:** Association of visceral fat area with Pre-frailty in logistic regression analysis

Variables	Crude	Model 1	Model 2	Model 3
	Crude OR (95% CI)	Adjusted OR (95% CI)		
Age	1.02 (0.96 − 1.09)	1.04 (0.97 − 1.03)	1.01 (0.94 − 1.08)	1.02 (0.95 − 1.09)
Sex (women)	0.84 (0.41 − 1.74)	0.76 (0.36 − 1.61)	0.72 (0.33 − 1.54)	0.58 (0.26 − 1.31)
VFA ≥ 100 cm^2^	**2.60 (1.08 − 6.25)**	**2.95 (1.22 − 7.11)**	**2.96 (1.21 − 7.24)**	**3.15 (1.26 − 7.87)**
The number of prescription medications taken per day	**1.26 (1.06 − 1.50)**		**1.26 (1.05–1.51)**	**1.25 (1.04 − 1.49)**
Depression	**2.28 (1.05 − 4.94)**		**2.01 (0.93–4.36)**	**2.28 (1.02 − 5.11)**
Going out less than last year due to the COVID-19	**3.03 (1.46 − 6.28)**			**3.46 (1.55–7.74)**

*Abbreviations: OR* Odds ratio, *CI* Confidence interval, *VFA* Visceral fat area

Model 1: Adjusted for sex and age. Goodness-of-fit: H‐L Chi^2^ (8) = 6.8716, *p* = 0.568

Model 2: Adjusted for sex, age, the number of prescription medications taken per day, and depression. Goodness-of-fit: H‐L Chi^2^ (8) = 1.128, *p* = 0.997

Model 3: Adjusted for sex, age, the number of prescription medications taken per day, depression, and going out less than last year due to the COVID-19. Goodness-of-fit: H‐L Chi^2^ (8) = 6.449, *p* = 0.597

## Discussion

In this study, we found a relationship between visceral fat accumulation and pre-frailty. Older adults with high VAF are at increased risk of pre-frailty after adjusted for age, sex, health status, and impact of the COVID-19 pandemic. It is worth noting that 6.5% of women were observed that have no BMI overweight but have high VFA. This study suggests the importance of considering VFA for the prevention of pre-frailty among community‐dwelling older people.

A higher prevalence of pre-frailty is noted during the COVID-19 pandemic. A previous online survey on 1,600 Japanese community-dwelling older adults in April 2020 has reported that 40% of participants had pre-frailty [9]. However, in this study, there are 75% of participants were pre-frailty in November 2020. As the social distancing or stay-at-home time becomes longer and longer, the physical activity of older adults also decreased. Moreover, the assessment scales are different, and the participants measured are different. The previous research is an online survey, so it is impossible to confirm the daily activity ability of the participants. However, this research was done in the elderly welfare center, and the participants were all independent. Taking into account the above reasons may have led to the higher prevalence of pre-frailty in this study. As pre-frailty had a strong impact on the risk of future frailty and disability. The early detection and prevention of frailty are important for community-dwelling older adults with a pre-frailty.

Daily physical activity behaviors can have an impact on visceral adipose tissue [29]. Going out less than last year is frequently observed in community‐dwelling older people during the COVID-19 pandemic. Due to the COVID-19 epidemic, a previous study has reported total physical activity time per week for older adults decreased by 65 min [9]. In this study, we found that 78% of older adults have reduced going out due to the COVID-19 pandemic. The negative consequences of the decrease in physical activity observed during COVID-19 affect both physical and mental health. On one hand, lower physical activity levels lead to lower energy expenditure and a higher risk of overweight and obesity [30]. Thus, the combined effect of physical inactivity and additional stress during COVID-19 may lead to an increase in fat accumulation. A previous study showed that the most prevalent unhealthy change in behavior was 32% of participants increased food intake, and 29.4% of participants increased weight during the Lockdown in the United Arab Emirates. The female gender, living in an apartment and being overweight or obese were more likely to report unhealthy lifestyle changes [30]. In fact, staying home for a prolonged period may lead to more prevalent sedentary behavior. Sedentary behavior is considered to be a determining factor in obesity [31], and sedentary behavior affects muscle physiology that increasing the risk of sarcopenia [32]. Moreover, the co-existence of adiposity and low muscle strength is associated with sarcopenic obesity [33]. Thus, these results indicated that Japanese community‐dwelling older adults during the COVID-19 pandemic should take care of not only the accumulation of visceral fat but also the reduction of skeletal muscle mass. However, only a small number of older adults came to the center to participate in sports due to the social distancing of COVID-19. Thus, the situation of those who did not go out at home may be more serious.

In this study, high VFA was found to be a risk factor for pre-frailty. The impact of the pandemic on physical activity and lifestyle changes may lead to self-reported physical function decline. Moreover, it is well known that many diseases of older adults are related to frailty. Previous studies have shown that VFA increase the risk of obesity-related disorders. The proportion of subjects with multiple risk factors was significantly higher in obese subjects with a high VFA. Furthermore, the non-obese subjects with a high VFA showed a high prevalence of obesity-related disorders than non-obese subjects with normal VFA [34]. A better understanding of the relationship between visceral fat and pre-frailty is important for the prevention of frailty in older adults due to pre-frailty is potentially reversible. Future studies are needed to determine the causal relationship between VAF and pre-frailty through longitudinal studies. Although the portable BIA machine is easy to measure the VFA in the community setting [35], previous studies have reported a strong association between visceral fat and waist-to-hip ratio (WHR) [34]. Because measuring WHR is a simple, convenient, and inexpensive tool that can be used as a surrogate for measuring VFA to convenient screening for pre-frailty. Thus, the association between visceral fat accumulation and premature aging may help identify new prevention targets. It is worth noting that 6.5% of women were observed that have no BMI overweight but have high VFA among these VAF overweight people. A previous study reported that 18.6% of people are not obese but have high VFA, and high VFA is associated with an increase in the number of complications [36]. A previous study comparing Asian ethnic Caucasians have reported that Asians have a higher body fat deposition at a lower BMI than Caucasians [34]. Abdominal obesity promotes insulin resistance and leads to metabolic syndrome [37]. Therefore, at a relatively low BMI, Asians have higher glucose intolerance and cardiovascular risk factors compared to Caucasians [34]. Further studies are necessary to define obesity to predict frailty risk among older adults, especially among relatively lean older adults, and to determine the different outcomes of different obesity in older adults.

There are some limitations in this study that should be considered in future studies. First, the elderly welfare centers had reopened for use during the time of this study’s investigation, but group classes had been suspended due to the COVID-19 infection prevention measure. Thus, the measure of frailty in this study used a subjective screening scale, and future studies will need to reduce the bias of subjective measures through objective measures. Second, our study included participants who were attendees of welfare centers (a social facility for older adults) in Sapporo, Hokkaido, a region that has been heavily impacted by the COVID-19 pandemic in Japan. It is worth noting that only a small number of older adults came to the center to participate in sports due to the social distancing of COVID-19. Thus, most participants were independent, and this finding may not be reflective of the general older Japanese population. Likewise, we only focused on evaluating participants who came to welfare centers. Thus, people did not come to the centers due to COVID-19 being unable to be collected. Secondly, this study is a cross-sectional study and cannot determine the VFA of the elderly before the COVID-19 epidemic, nor the causal relationship between VFA and pre-frailty. Therefore, further prospective studies are needed to identify mechanistic links between VFA and pre-frailty in this population. Finally, as with any study, results may be subject to confounding bias from other unknown variables.

In conclusion, our study has found that 78% of older adults have reduced going out due to the COVID-19 pandemic. There were 27.3% of participants with high VFA. It is worth noting that some older adults have healthy BMI but have high VFA, and are more likely to be women. And in the COVID-19 pandemic, 75% of the older adults have pre-frailty. Higher VFA is related to the increase in pre-frailty, even after adjusted for age, sex, health status, and impact of the COVID-19 pandemic. The association between visceral fat accumulation and pre-frailty may help identify new prevention targets. Further longitudinal studies are needed to determine their mechanisms in this population. However, for the older adults who have not been able to come to the center due to the social distance of COVID-19, their living habits and physical conditions may be more affected. It reminds the importance of home exercise and diet guidance for community-dwelling older adults to prevent muscle loss and associated physical decline in community-dwelling older adults during the COVID-19 pandemic.

## Data Availability

The datasets generated and/or analysed during the current study are not publicly available due to its ethical concerns, supporting data cannot be made openly available, but are available from the corresponding author on reasonable request.
